# Transformer-encoded nnU-Net with local region perceptron and contrastive learning (TLC-nnUNet) for multiple brain metastasis detection and delineation

**DOI:** 10.1088/1361-6560/ae4ced

**Published:** 2026-03-13

**Authors:** Kangning Zhang, Gregory Arthur Szalkowski, Scott Soltys, Yusuke S Hori, David J Park, Robert Timmerman, Zabi Wardak, Lili He, Qingying Wang, Mingli Chen, Hao Jiang, Weiguo Lu, Xuejun Gu

**Affiliations:** 1 Department of Radiation Oncology, Stanford University, Stanford, CA 94305, United States of America; 2 Department of Neurosurgery, Stanford University, Stanford, CA 94305, United States of America; 3 Department of Radiation Oncology, The University of Texas Southwestern Medical Center, Dallas, TX 75390, United States of America; 4 Cincinnati Children’s AI Imaging Research (CAIIR) Center, Cincinnati, OH 45229, United States of America

**Keywords:** brain metastases detection and segmentation, vision transformer, contrastive learning, supervised pretraining

## Abstract

Accurate detection and segmentation of multiple brain metastases (BMs) on magnetic resonance image remain challenging, particularly for those involving small lesions (longest axis length <3 mm), due to limitations in sensitivity, precision, and feature representation in existing deep learning frameworks. To address this challenge, we develop TLC-nnUNet, a novel integration of two advancements into transformer-enhanced nnU-Net (T-nnUNet) architecture: (1) local region perceptron (LRP), a loss constraint prioritizing small BM detection by up-weighting underrepresented voxels; and (2) contrastive learning pretraining (CLP), a supervised model pre-training strategy to amplify latent-space divergence between BM and non-BM regions, reducing false positives (FPs). The developed TLC-nnUNet is trained and evaluated on a multi-institutional dataset and achieves state-of-the-art performance, with 89.70% sensitivity, 97.34% precision, and a Dice coefficient of 0.92 at patient level. Further ablation studies confirm the synergistic contributions of each component: LRP enhances small BM detection, while CLP refines feature contrast, reducing FPs. Visualization via t-distributed stochastic neighbor embedding underscores CLP’s role in disentangling BM and non-BM latent representations. Compared to existing methods, TLC-nnUNet demonstrates consistent accuracy of detection and segmentation cross lesion sizes. This framework holds significant promise for clinical workflows, enabling precise BM detection and segmentation in stereotactic radiosurgery and reducing manual contouring time.

## 1. Introduction

Brain metastases (BMs) occur in approximately 20%–40% of cancer patients and the onset is predominantly multiple (mBMs, Soffietti et al 2002). Historically, whole-brain radiotherapy (WBRT) was the standard of care for mBMs. While WBRT is effective, it can lead to cognitive decline (Chang et al 2009, Brown et al 2016). Stereotactic radiosurgery (SRS) has emerged as the preferred treatment, which precisely delivers highly focused radiation, effectively targeting the metastases while minimizing the damage to healthy brain tissue (Müller-Riemenschneider et al 2009, Arvold et al 2016, Mori et al 2022). The efficacy of SRS in BMs treatment relies on accurate BM detection and precise delineation (a.k.a segmentation) (Müller-Riemenschneider et al 2009, Steeg et al 2011). Conventionally, BMs are manually identified and delineated on T1-contrast (T1c) magnetic resonance image (MRI), a process that is labor-intensive and time-consuming. During SRS planning, the small ones are sometimes missed due to human oversights, particularly in cases involving mBMs (Zhou et al 2020b, Ziyaee et al 2023); and the large ones are commonly subject to inconsistent delineation due to inter-observer variation. Automatic mBMs detection and delineation (Pennig et al 2021, Ziyaee et al 2023) has the potential to reduce human oversights and eliminate inter-observer variations.

In recent years, deep learning (DL) models have demonstrated remarkable capabilities in medical image analysis, excelling in tasks such as classification, detection and segmentation (Shen et al 2017, Cai et al 2020, Suganyadevi et al 2022). In the context of image segmentation, U-Net architecture (Ronneberger et al 2015), a symmetrical U-shaped convolution neural network (CNN) structure, has established itself as a cornerstone for highly accurate image segmentation. The high performance of U-Net in segmentation is achieved through a contracting path that captures context and an expansive path that enables precise localization, along with skip connections that combine feature maps from both paths. Building on the U-Net, two key advancements have significantly improved segmentation performance: the automated pipeline optimization enabled by nnU-Net and the incorporation of transformer modules for global context modeling. The nnU-Net set a new state-of-the-art for segmentation tasks by systematizing and automating the complex process of manual configuration, which was previously addressed either by cumbersome tuning or purely empirical approaches with practical limitations. Even when nnU-Net underperforms in specialized domains, it serves as a robust foundation for further optimization. The transformer architecture, initially developed for natural language processing tasks (Vaswani 2017), has gained increasing popularity in the domain of image processing (Khan et al 2022). The key advantage of the transformer is its capability to model the global context using a self-attention mechanism. This is particularly suitable for overcoming the shortcomings of CNN-based U-Net, which struggles to model long-range relationships due to the intrinsic locality of convolution operations and experiences performance degradation when segmenting structures that vary in texture, shape, and size. Vision transformer (ViT) (Dosovitskiy 2020, Han et al 2022), known for its powerful global context modeling using self-attention and its flexible, modular architecture adaptable for downstream tasks, has been incorporated into U-Net and nnU-Net to form the TransUNet and later nn-TransUNet (Chen et al 2021a, 2024, Zhao et al 2022). In this paper, we denote nn-TransUNet as T-nnUNet to ensure consistency with our proposed model’s nomenclature.

Despite improvements in automated segmentation in medical image in general, a critical unmet need in the SRS clinical workflow remains the reliable detection of small size lesions (longest axis length <3 mm), which are particularly prone to being missed during manual reviews, especially in cases involving extensive metastatic burden. Note, in this paper we use longest axis length to categorize BMs size, with <3 mm as small, [3, 6] mm as medium, and >6 mm as large. In SRS planning, even small BMs may be treated if they are deemed progressive or symptomatic, and missing them can lead to undertreatment, potential local failure, or unanticipated neurological decline. Therefore, a system that increases the sensitivity of detecting these small size lesions without a substantial increase in false positive (FP) could significantly augment clinical decision-making and planning accuracy. Although T-nnUNet demonstrates high performance in general image segmentation tasks, its direct application in BMs, which requires distinct yet interdependent detection and delineation steps, may lead to compromised accuracy due to methodological incompatibilities. First, detecting small size BMs requires a model with high sensitivity to identify abnormal regions comprising only a few voxels while delineating large BMs demands a model with high accuracy of catching the intensity changes at the lesion boundaries. T-nnUNet, which uses a loss combining cross entropy (CE) and Dice coefficient (DC), could fail in detecting small size BMs: (1) CE is generally based on the Bernoulli distribution and works best with an equal data distribution among classes. Detecting small size BMs from a whole large-volume image introduces significant class imbalance, which may not be adequately evaluated by CE; (2) DC is inaccurate in measuring small volume overlap due to its numerical instability at small volumes; (3) T-nnUNet inherits the transformer’s data-hungry characteristic, requiring a large dataset for effective training. In the case of a lack of large datasets in medical imaging, the models might have to be degraded into 2D with inferior performance (Chen et al 2021a, 2024) or to be pre-trained with another available large-size dataset with implementation complexity.

To address these challenges and key bottlenecks in SRS workflow, we propose TLC-nnUNet, a clinically oriented AI framework that integrates a local region perceptron (LRP) and contrastive learning–based pretraining (CLP) within the T-nnUNet architecture. LRP is designed to boost detection of small BMs, while CLP curbs false-positive detections for small lesions and enhances delineation accuracy for larger BMs. Our goal is to deliver an AI tool that combines technical innovation with real-world applicability in time-pressure SRS settings, where lesion detectability, consistency, and segmentation precision are paramount. Specifically, our work makes three major contributions:

- To improve the small BMs detection rate, we designed LRP that analyzes the MRI in small 3D regions (local patches) and gives higher weight to areas containing small or hard-to-detect lesions. Additional weighed LRP loss $\left(L_{\text{LRP}}\right)$ term except the conventional $L_{\text{Dice}}$ and $L_{\text{CE}}$ terms (see equation (1)) employs a focal loss (Lin et al 2017) to address the class imbalance in small size BMs detection.
- To address the data-demanding issue in training transformers, we develop CLP, a pretraining step, which is designed to accentuate the contrastiveness of latent features between regions with and without BMs using a supervised learning technique. Further, we use t-distributed stochastic neighbor embedding (t-SNE) visualization to demonstrate that the latent representations of regions with and without BMs are more distinguishable after CLP.
- We demonstrate in extensive experiments that TLC-nnUNet achieves higher sensitivity and precision for BMs detection and higher accuracy for BMs delineation compared to state-of-the-art methods.

## 2. Methods and materials

### 2.1. Overview

**Figure 1.**
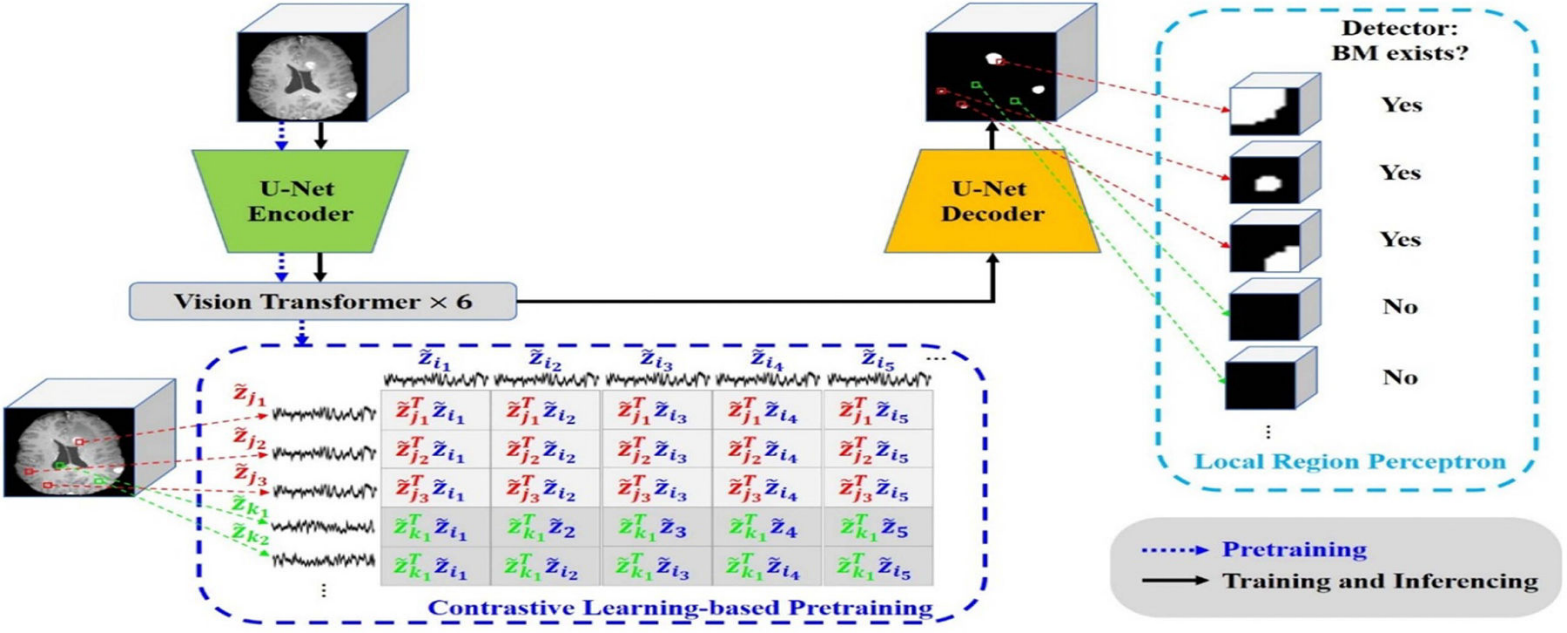
Schematic of TLC-nnUNet for BMs segmentation, which includes a CLP pipeline, T-nnUNet architecture and a constraint of LRP.

The overall framework of TLC-nnUNet, illustrated in figure 1, comprises three main components: the T-nnUNet model, the loss constraint with LRP, and the supervised CLP. The model’s input is a 3D T1c MRI and outputs a binary-mask image with 1 and 0 indicating BM and non-BM voxels respectively. Here we chose T1c-only inputs to match common clinical protocols, where T1c imaging is the primary and/or the sole sequence for SRS treatment and follow-up. The encoder, incorporating downsampling, processes the input to extract high-dimensional features. Then, a 6-layer ViT analyzes the features extracted from the encoder via a patch-by-patch self-attention mechanism. The ViT constructed visual representation is reshaped and embedded into the up-sampling decoder of U-Net architecture produces the output. The loss constraint with LRP (section 3.2) is designed to up-weigh the small BMs in local regions to enhance the detectability of small BMs. A contour labeling-free pretraining process, CLP (section 3.3), is developed, which accentuates the differences between voxels inside and outside BMs. As named, TLC-nnUNet is built on nnU-Net framework. Similar to nnU-Net (Isensee et al 2021), TLC-nnUNet includes experimental planning and preprocessing steps, which uses the heuristic rules and conducts automatically.

### 2.2. LRP constrained loss function

For segmentation tasks in DL, we adopt the combination of Dice loss and CE loss (Isensee et al 2021) as the basic loss function with their high performance in general segmentation tasks with $L=L_{\text{Dice}}+L_{CE}$. To enhance the performance of T-nnUNet for detecting small BMs, we incorporate an LRP loss term in addition to Dice and CE losses in the overall model loss function. Since both CE loss and LRP loss are classification-oriented, they operate on predicted class probabilities and directly encourage correct class assignments. Therefore, it is natural to combine and weight LRP with CE loss using the parameter w, as both losses reinforce each other in guiding the model’s class-level predictions. The loss function is as follows:

(1) $$\begin{array}{rr} & L=L_{\text{Dice}}+(1-w)L_{CE}+wL_{LRP}\\ & \text{with}\;L_{\text{Dice}\;}=\frac{2\sum_{i\in I}\;\;{se}_{pred}^{i}{seg}_{GTT}^{i}}{\sum_{i\in I}\;\;{seg}_{pred}^{i}+\sum_{i\in I}\;\;{seg}_{GT}^{i}},\\ & \text{and}\;L_{CE}=-\sum_{l}^{C}\;\;x_{l}log\left(p_{l}\right),L_{LRP}=-\sum_{l=1}^{C}\;\;y_{l}{\left(1-q_{l}\right)}^{\gamma }log\left(q_{l}\right). \end{array}$$

Here the term $L_{\text{Dice}}$ represents the standard Dice loss, which calculates the voxel-wise overlap between the prediction of TLC-nnUNet, ${seg}_{\text{pred}}$ and ground truth ${seg}_{GT}$. The $L_{CE}$ teram is a standard crossentropy loss used for global classification, where $x_{l}$ is the binary ground truth and $p_{l}$ is the predicted probability for the correct label.

The LRP loss ($L_{\text{LRP}}$) is a patch-based focal loss term specifically designed to address the small BMs detection problem under extreme foreground–background imbalance. We first divide the entire 3D volume (e.g. 256 × 256 × 256) into a series of 8 × 8 × 8 patches (figure 1). Each patch l is assigned a local ground truth label $y_{l}\in \{0,1\}$. A patch is automatically labeled $y_{l}=1$ if it contains at least one voxel of a BM; otherwise, it is labeled $y_{l}=0$. To handle the extreme imbalance between patches that contain lesions and the vast number of empty background patches, we apply a focal weight in the $L_{\text{LRP}\;}$ term, with $q_{l}$ as the predicted probability of a lesion presence in path l and γ is a focusing parameter (set to 0.5 in this study) that down-weights the influence of easy negative patches (background) to focus the gradient on hard positive regions. The contribution of the LRP loss is controlled by w, a hyperparameter representing the weight of the LRP term. Through empirical testing in section 3.6, we identified w=0.4 as the optimal value for balancing sensitivity and precision.

### 2.3. A CLP-enabled pre-training strategy

We propose a supervised CLP pipeline to address the challenge of BM segmentation, particularly under limited contour-annotated data. By learning discriminative latent representations, CLP enhances the separability between voxel-level regions containing BMs and healthy tissue while preserving mutual information among regions with similar BM characteristics. This approach improves model generalizability by accentuating feature contrast in the encoder’s latent space, effectively acting as a pretraining strategy to prime the network for downstream segmentation tasks. CLP builds on its proven success in image classification (Wang et al 2021, Chen et al 2021b) and medical applications where a significant imbalance exists in labeling conditions (Alam et al 2023). For BMs segmentation, we adopt CLP to explicitly amplify divergence between BM-positive and BM-negative regions, ensuring robust feature learning even with classification annotations only. This pretraining phase not only mitigates performance degradation from limited labels but also establishes a foundation for high-precision delineation in clinical workflows.

In pretraining step, unlike SimCLR (Chen et al 2020), where the data augmentation (rotation, translation, etc) are conducted on image patch before feeding into neural network, CLP augments the data on its latent representation. We extract latent representation z by the base encoder $f(\cdot ),z=f(x)\in R^{d\times L}$, which is the output of the ViT layer with a feature dimension of d.L is the length of the latent information equals to the size of 8^3^, a 3D subsampled input. In the data augmentation step, we first vectorize the BMs binary mask M, denoted as $\tilde{M}\in R^{L}$,with a 8^3^ 3D subsampling. Then, we augment the latent vector z into ${\tilde{z}}_{i}$ and ${\tilde{z}}_{j}$ considered as positive pair if and only if ${\tilde{M}}_{i}={\tilde{M}}_{j}=1$; meanwhile, ${\tilde{z}}_{i}$ and ${\tilde{z}}_{k}$ considered as negative pair if and only if ${\tilde{M}}_{i}\neq {\tilde{M}}_{k}=0$, where i,j and k are the index of the vectors entries. Finally, we adopt the InfoNCE loss (Oord et al 2018) to explicitly optimize the similarity between positive pairs while contrasting them against negative pairs. Formally, the optimization is expressed in equation (2):

(2) $$\underset{\left({\tilde{z}}_{i},{\tilde{z}}_{j}\right)}{argmax}E\left[log\frac{g\left({\tilde{z}}_{i},{\tilde{z}}_{j}\right)}{\sum_{j}\;\;g\left({\tilde{z}}_{i},{\tilde{z}}_{j}\right)}\right]+\underset{\left({\tilde{z}}_{i},{\tilde{z}}_{k}\right)}{argmin}E\left[log\frac{g\left({\tilde{z}}_{i},{\tilde{z}}_{k}\right)}{\sum_{k}\;\;g\left({\tilde{z}}_{i},{\tilde{z}}_{k}\right)}\right]$$

where g is an exponential function measuring the similarity between two vectors and E represents the expectation.

**Table:** 

** Algorithm 1.** Pseudo-code of CLP algorithm.
** inputs:** image x, subsampled BMs mask $\tilde{M}\in R^{L}$, encoder $fz=f(x)\in R^{d\times L}$
**for all** $i\in \left\{i|M_{i}=1\right\}$ **do**
**for all** $j\in \left\{j|M_{j}=1,j\neq i\right\}$ **do**
**for all** $k\in \left\{k|M_{k}=0\right\}$ **do**
${\tilde{z}}_{i}\in R^{d\times 1},$ ${\tilde{z}}_{j}\in R^{d\times 1},$ ${\tilde{z}}_{k}\in R^{d\times 1}$
**define** $\ell (i,j)=exp\left({\tilde{z}}_{j}^{T}{\tilde{z}}_{i}\right)$ **and** $\ell (i,k)=exp\left({\tilde{z}}_{k}^{T}{\tilde{z}}_{i}\right)$ **end for**
**end for**
$L=\sum_{\left(i,j),(i,k\right)}\;\left(-log\frac{\ell (i,j)}{\sum_{j}\;\ell (i,j)}+log\frac{\ell (i,k)}{\sum_{k}\;\ell (i,k)}\right)$
**end for**
**Return** encoder f

Thus, we can define the loss function with equation (3):

(3) $$L_{CLP}=\sum_{\left(i,j),(i,k\right)}\;\left\{-log\frac{exp\left({\tilde{z}}_{j}^{T}{\tilde{z}}_{i}\right)}{\sum_{j}\;I_{\left[j\neq i\right]}exp\left({\tilde{z}}_{j}^{T}{\tilde{z}}_{i}\right)}+log\frac{exp\left({\tilde{z}}_{k}^{T}{\tilde{z}}_{i}\right)}{\sum_{k}\;exp\left({\tilde{z}}_{k}^{T}{\tilde{z}}_{i}\right)}\right\}$$

where ${\tilde{z}}_{j}^{T}{\tilde{z}}_{i}$ represents the correlation between the two inputs to measure the similarity between the vectors ${\tilde{z}}_{i}$ and ${\tilde{z}}_{j}.\;I_{\left[j\neq i\right]}\in Error!$ Bookmark not defined. is an indicator function evaluating to 1 if and only if j≠i. Algorithm 1 summarizes the proposed CLP method.

### 2.4. Training

The proposed TLC-nnUNet model is trained with a fixed total number of 8000 epochs, with each epoch consisting of 201 batch iterations. The input of TLC-nnUNet is a 3D MRI image while the output is 3D binary mask with the same size. The network is trained using a stochastic gradient descent optimizer with Nesterov momentum set to 0.99. A polynomial learning rate schedule is employed, starting with an initial learning rate of 0.001.

Among these 8000 epochs, we use 4000 epochs for pretraining the encoder with ViTs while freezing the decoder, with the InfoNCE loss to optimize the latent representation. Following the pretraining, we fix the encoder and ViTs parameters and only fine-tune the decoder with 4000 epochs by using the loss function described in equation (1). Freezing the encoder during fine-tuning provides several practical benefits. First, it preserves the pretrained feature representations and mitigates catastrophic forgetting, which leads to more stable and efficient convergence than training the entire model in this stage. Second, it enhances data efficiency by reducing the number of trainable parameters, which is particularly advantageous when annotated datasets are limited. Finally, the fixed encoder facilitates interpretability study, as the learned latent representations can be analyzed without influence brought by training the decoder.

## 3. Experiments and results

### 3.1. Dataset

**Figure 2.**
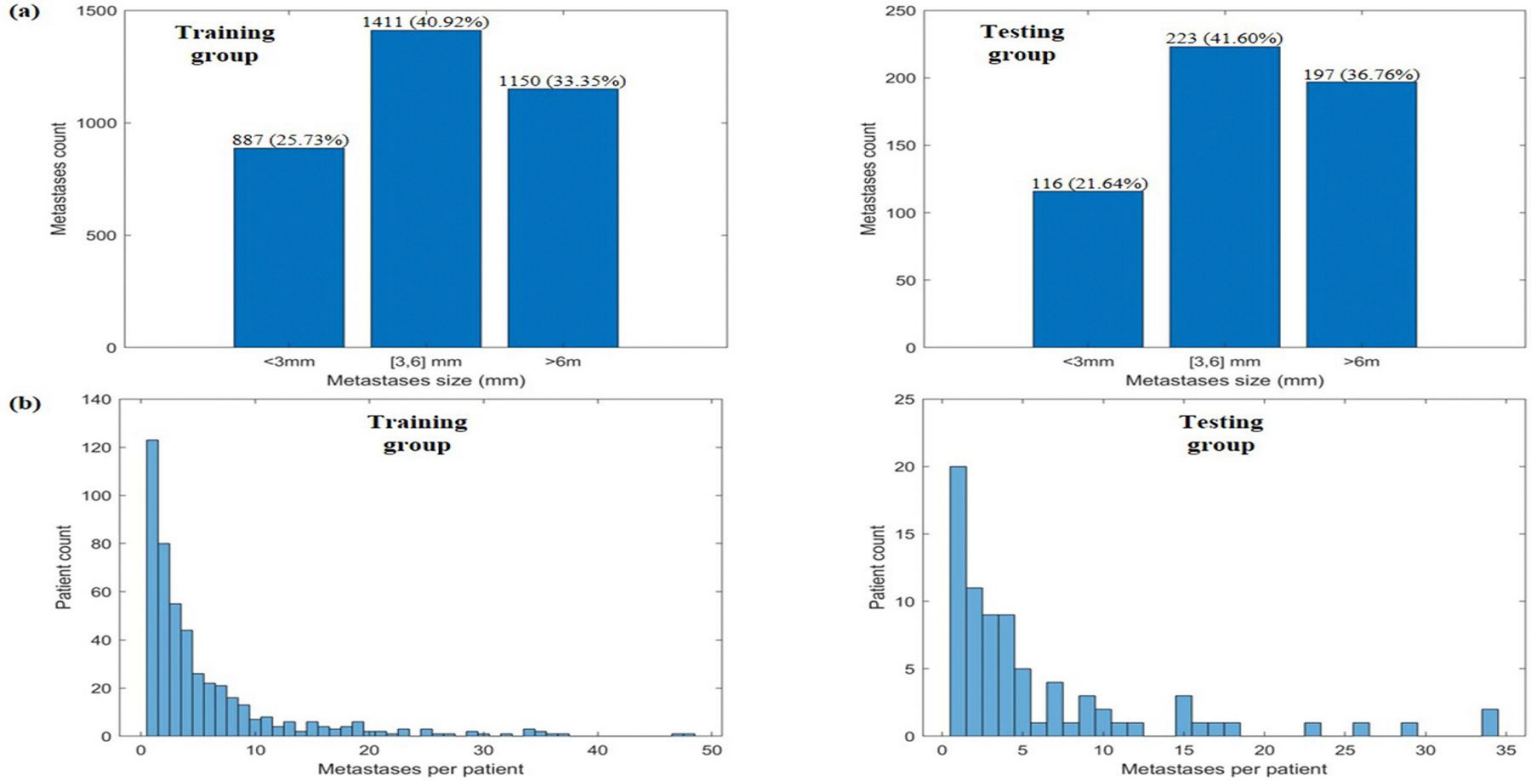
(a) Bar charts display the distribution of BMs sizes in the training group (left) and the testing group (right). (b) Bar charts illustrate the distribution of BMs across patients in the training group (left) and the testing group (right).

In this study, we employ both BraTS-METS 2023 dataset (Moawad et al 2024) and UTSW dataset (Yang et al 2020) for the model training and testing. BraTS data provides three labels: (1) Gd-enhancing tumor (ET—label 3), surrounding non-enhancing FLAIR hyperintensity (SNFH—label (2), and the non-enhancing tumor core (NETC—label 1). We combined label 1 (NETC) and label 3 (ET) to form the BMs delineation ground truth (GT). For UTSW data, we directly used clinically treatment target volume as GT. The combined dataset comprises a total of 481 patients (238 BraTS patients and 243 UTSW patients)), each with one axial 3D T1c MRI. Following the nnU-Net data management strategy, the dataset are randomly partitioned into a held-out test set [õne-sixth of the data, 79 patients (39 BraTS patients and 40 UTSW patients)] and a development cohort [five-sixths, 402 patients (199 BraTS patients and 203 UTSW patients)], with the latter undergoing 5-fold cross-validation for model training and evaluation. Within the nnU-Net framework, hyperparameters were automatically optimized based on the training data characteristics and cross-validation performance. During data collection, no restrictions were imposed on the size and/or the number of BMs. The distributions of BM sizes and the number of BMs per patient in both the training and test groups are illustrated in figure 2. As shown in figure 2(a), the majority of BMs in the training dataset have a size (longest axis length) within the [3, 6] mm range (1411, 40.92%), followed by those >6 mm (1150, 33.35%) and those <3 mm (887, 25.73%). A similar trend is preserved in the testing set, where the 3–6 mm category contains the highest number of BMs (223, 41.60%), followed by >6 mm (197, 36.76%) and <3 mm (116, 21.64%). Figure 2(b) illustrates the distribution of BMs per patient in the training (left) and testing (right) groups. In both sets, the majority of patients have a relatively small number of BMs, with the number of patients decreasing as the number of BMs increases. The training group exhibits a broader range of BM numbers, with some patients having up to 50 metastases, while the testing group has a lower overall count, with a maximum of ~35 metastases.

### 3.2. Evaluation metrics

The predicted lesion volume was formed with connected positive predicted voxels. A morphological closing operation with a 1 mm kernel was applied to the network output voxels to reduce small gaps and noise. Connected-component analysis using 26-neighborhood connectivity was then performed to group voxels into predicted lesion volumes. The performance of the proposed TLC-nnUNet was evaluated with three metrics (1) sensitivity and (2) precision for BM detection, and (3) DC for BM segmentation. For detection, we computed 3D coverage (CV) between each predicted volume (A) and GT volume (B) pair on the image grid ($CV=\frac{\left|A\cap B\right|}{\left|B\right|}$. Each predicted volume needs to be evaluated with all GTs. The prediction was considered as a true positive (TP) and matched to a GT lesion if CV ⩾ 0.5. Predicted volumes not matched to any GT lesion (B=0) were counted as FPs and GT lesions with no matched prediction (A=0) were counted as false negatives (FNs). Sensitivity and Precision were calculated as $\frac{TP}{TP+FN}$, and $\frac{TP}{TP+FP}$, respectively. Lesion size was defined from the longest 3D diameter of GT mask. DC was calculated only for each TP lesion with its matched GT lesion size ⩾3 mm (i.e. the medium and large two strata combined), Thus, DC were not evaluated for FP and FN lesions and GT lesions <3 mm. The defined three metrics were reported at both BMs level and patient level. At the BMs level, sensitivity and precision were computed per size strata (small <3 mm, medium [3–6] mm and large >6 mm); and mean DC was computed by averaging per-lesion DC over two strata combined. At the patient level, per-patient sensitivity and precision were computed across all lesions within the patient without size stratification. And per-patient DC was calculated by averaging per-lesion DCs within the patient.

To evaluate the effectiveness of CLP, we employed t-SNE (Hinton and Roweis 2002) to visualize the latent representations from the output of ViT layer output of the T-nnUNet and TC-nnUNet. The t-SNE is a statistical method for visualizing high-dimensional feature data by giving each datapoint a location in a two or three-dimensional map. In the context of BMs detection and segmentation, it focuses on the divergence distance between the latent representation of voxels inside and outside of BMs.

### 3.3. Performance comparison with state-of-the-art methods

Table 1 lists the performance comparison between our TLC-nnUNet with state-of-the-art BM detection and segmentation on T1c MRI, reported across lesion size categories. The metrics evaluating our TLC-nnUNet performance are reported at BMs level. Our TLC-nnUNet achieves the highest segmentation accuracy (mean DC = 0.90) on the pooled data, surpassing prior methods (0.67–0.89). Its detection precision in pooled testing data is strong and scales with lesion size (82% <3 mm, 92% 3–6 mm, 99% >6 mm), outperforming Asymmetric UNet (Cao et al 2021) and DeSeg (Yu et al 2023) across bins and greatly exceeding SSD for medium and large lesions (92% vs 35% and 99% vs 36%). Sensitivity is balanced (66%/88%/94% for small/medium/large), markedly higher than SSD for small and medium lesions (66% vs 15% and 88% vs 70%) and competitive for large lesions (94% vs 98%). While some methods report high overall sensitivity without size stratification (e.g. ~94%–95%), TLC-nnUNet provides stratified performance, demonstrating robust detection and state-of-the-art segmentation across lesion sizes. We also conducted additional analysis using a sub-cohort of the mixed testing dataset. As shown in table 1, the precision, sensitivity, and mean DC of the sub-cohort are similar to those of the mixed evaluation dataset. Furthermore, to demonstrate our model’s generalization across different datasets, we conducted cross-dataset experiments. Specifically, we trained and tested two more models: one trained on BraTS data and tested on UTSW data and the other trained on UTSW data and tested on BraTS data. Overall, the results from cross-data experiments are similar to the pooled data, implying the generalizability of the trained models. Notice the detection and segmentation of BMs primarily rely on the enhanced Gadolinium contrast image feature on T1 MRI, and this feature tends to exhibit minor variations among different institutions. Thus, that the model perform similar on different dataset could be attributed to the image-feature similarity across institutions.

### 3.4. Ablation studies at patient level

**Figure 3.**
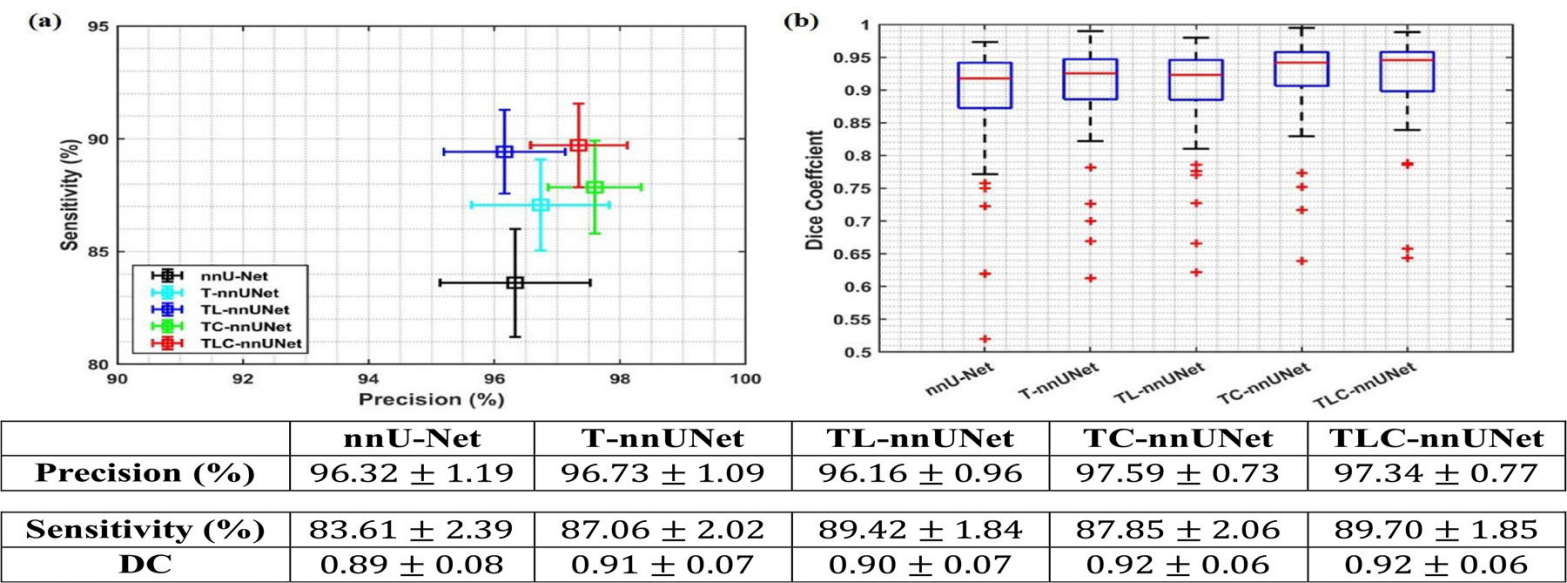
The performance of five different methods: nnU-Net, T-nnUNet, TL-nnUNet, TC-nnUNet, and TLC-nnUNet at patient level, (a) mean and standard deviation of sensitivity and precision across all sizes of BMs. (b) DC for BMs with the size of [3, 6] mm and >6 mm. The table below provides the corresponding values for the mean and standard deviation of sensitivity and precision across the five different methods.

We conduct ablation studies to assess the contribution of each component in our TLC-nnUNet by evaluating the performance of several model configurations, including nnU-Net, T-nnUNet, TL-nnUNet, TC-nnUNet, and TLC-nnUNet. The quantitative results are illustrated in figure 3. In terms of detection results, specifically precision and sensitivity, as illustrated in figure 3(a), T-nnUNet exhibits a 0.2% increase in precision and a notable 4.1% improvement in sensitivity compared to nnU-Net. Notably, TL-nnUNet that incorporates LRP with T-nnUNet achieves substantiable gains in sensitivity compared to T-nnUNet, albeit with a slight reduction in precision. In contrast, TC-nnUNet with CLP improve both precision and sensitivity compared to T-nnUNet. The TLC-nnUNet, which integrates LRP and CLP with T-nnUNet, achieves the best the optimal and balanced performance among all the models regarding both precision and sensitivity. Notably, the addition of supervised CLP provides TC-nnUNet with an additional improvement of approximate 2.1% in segmentation performance than T-nnUNet, and around 2% improvement for TLC-nnUNet compared with DC value by TL-nnUNet model, as illustrated in figure 3(b).

Overall, TLC-nnUNet, demonstrates superior performance, achieving a precision of 97.34 ± 0.77%, a sensitivity of 89.70 ± 1.85% and a DC of 0.92 ± 0.06 at patient level. These results underscore the model’s effectiveness in accurately identifying BMs while minimizing FNs.

**Figure 4.**
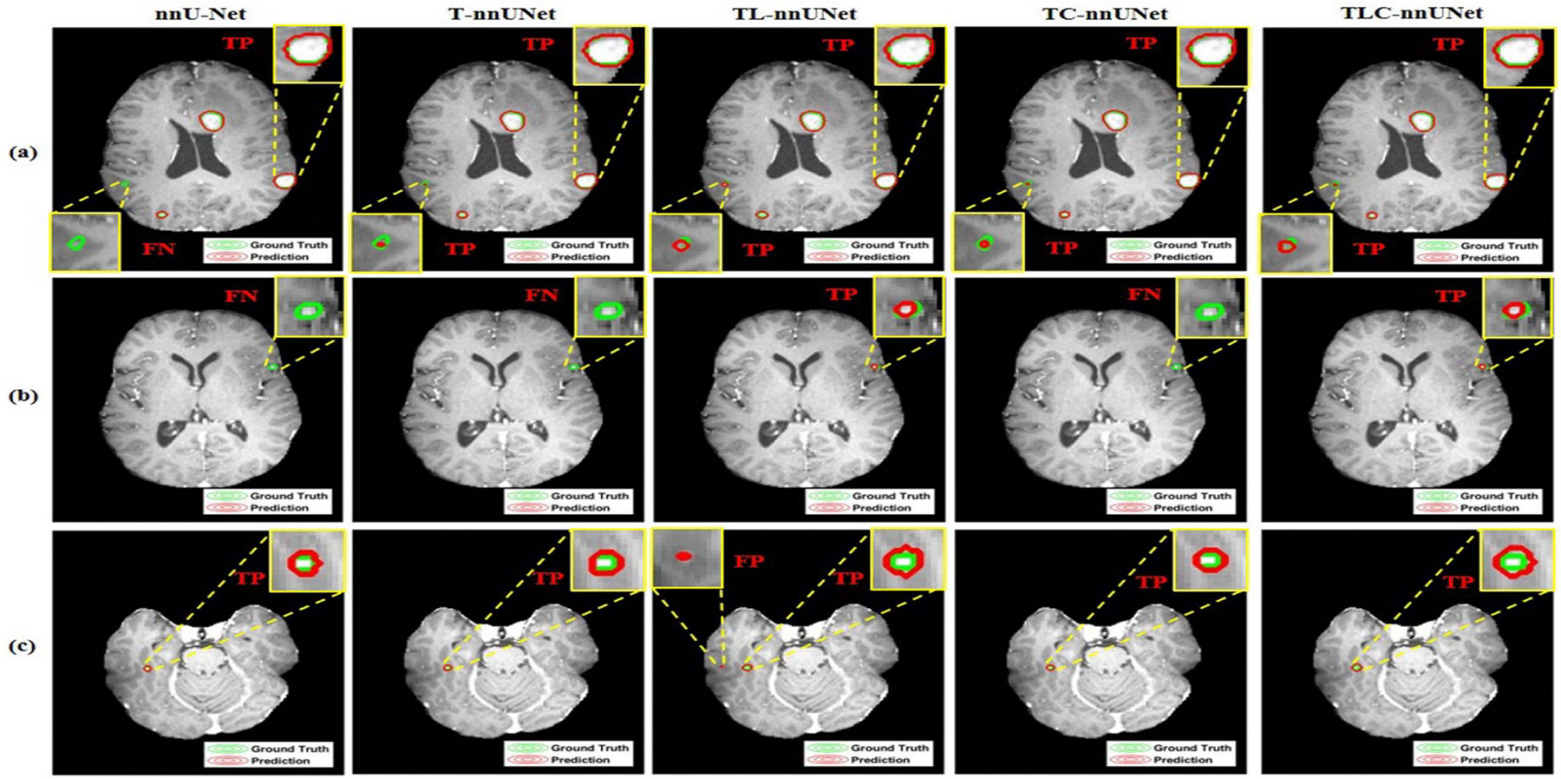
Examples of segmentations and detections from the nnU-Net, T-nnUNet, TL-nnUNet, TC-nnUNet and TLC-nnUNet. (a)–(c) Results of BMs segmentation accuracy with ground truth (green contour), prediction (red contour), and labels of TPs, FNs and FPs from three samples.

In addition, representative examples of segmentation, TP, FP, and FN predictions are illustrated in figure 4, illustrating the model’s behavior across different scenarios. As illustrated in figure 4(a), all five methods exhibit high segmentation accuracy for medium to large BMs (⩾3 mm) and the transformer-based methods consistently outperform nnU-Net on detecting the smaller target. Predicting small BMs (<3 mm) poses great challenges for nn-UNet, T-nnUNet, and TC-nnUNet as seen in figure 4(b). Because of introducing LRP, TL-nnUNet and TLC-nnUNet have high sensitivity and be capable of detecting small BMs in figure 4(b); however, LRP along could increase the risk on precision as seen in the TC-nnUNet in figure 4(c). Ultimately, TLC-nnUNet keeps a good balance on reducing FP and FN.

### 3.5. Analysis model’s detection capability with different BMs size

**Figure 5.**
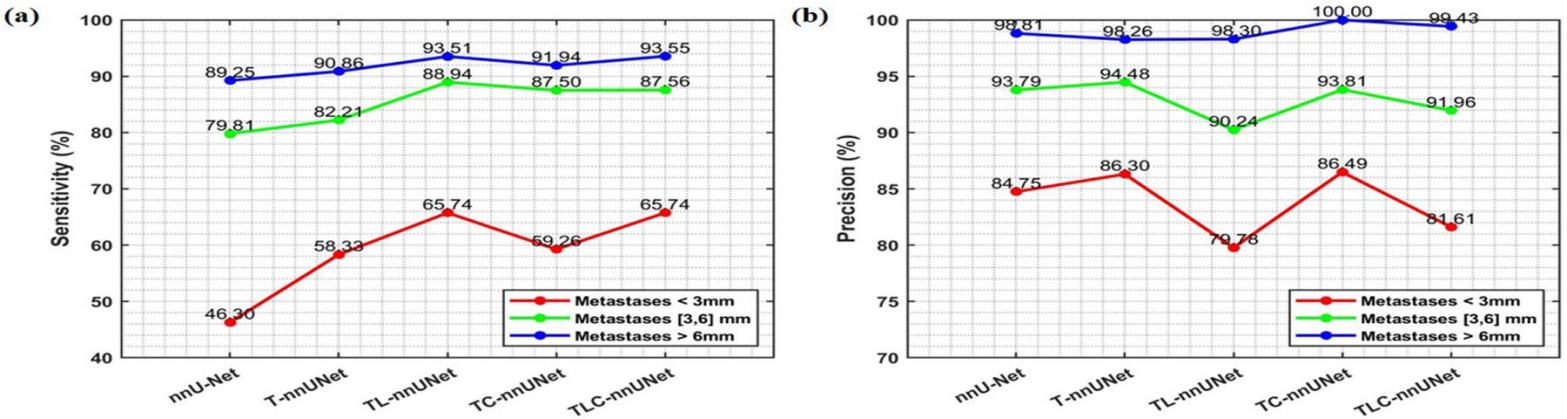
(a) Sensitivity performance of five different methods of nnU-Net, nnU-Net with LRP, nnU-Net with CLP, T-nnUNet, TL-nnUNet, TC-nnUNet, and TLC-nnUNet for BMs in different sizes (<3 mm, [3, 6] mm, >6 mm). (b) Precision performance of the five different methods for BMs in different sizes.

Detection results in precision and sensitivity of various models at BM level are summarized in figure 5. For small BMs, our results indicate that T-nnUNet significantly improves sensitivity, increasing from 46.30% to 58.33% (figure 5(a)) and enhancing precision from 84.75% to 86.30% (figure 5(b)). With the addition of LRP, TL-nnUNet achieves sensitivity of 65.74%, further improved 7.41% over T-nnUNet, but drops the precision down to 79.76%. With the addition of CLP, TC-nnUNet does barely improve sensitivity (+0.93%) and precision (+0.19%) compared to T-nnUNet. By combining both LRP and CLP, TLC-nnUNet achieves notable gains in sensitivity (+7.41%) with a slight compromising precision (−4.69%) compared to T-nnUNet. For medium and large BMs via all the models, similar patterns are observed, though the improvements are less pronounced than those for small BMs.

### 3.6. Impact of LRP weights at BM level

**Figure 6.**
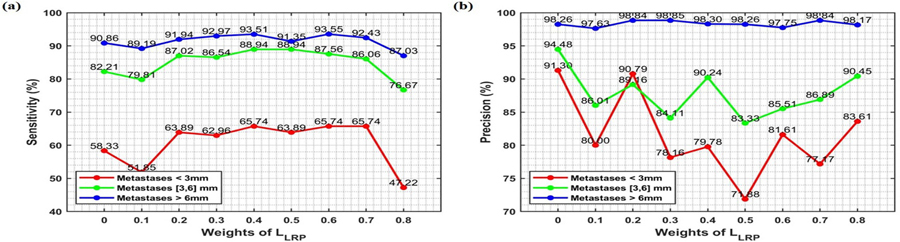
(a) Sensitivity and (b) Precision of TL-nnUNet under different weights for BMs in different sizes (<3 mm, [3, 6] mm, >6 mm).

As LRP is mainly designed for improving BM detection accuracy, we further investigate the impact of LRP weights in the loss function on detection precision and sensitivity at BM level. The results with varied weight *w* in equation (1) from 0.1 to 0.8 in increments of 0.1 are shown in figure 6.

For sensitivity, figure 6(a) illustrates an approximately convex curve as the LRP weight increases. At a small LRP weight (=0.1), the model lacks the sensitivity in detecting small BMs, resulting in low accuracy for sensitivity of 51.85%. Conversely, at a large LRP weight (=0.8), the model tends to bias toward FPs at the voxel level and negatively affects small BM detection, resulting in low sensitivity of 47.22%. For precision, there is no definitive relationship between precision and LRP weight; however, a weight of 0.2 appears optimal in terms of precision.

Overall, applying LRP shows enhancing sensitivity, but may reduce precision. Thus, selecting an appropriate LRP weight is crucial for achieving optimal predictions. For this study, an optimal weight of 0.4 was identified based on sensitivity analysis (figure 6(a)), yielding a sensitivity of 65.74% for small BMs, 88.94% for medium size, and 93.51 for large size.

### 3.7. The t-SNE visualization of latent representation

To investigate how the CLP improves precision and sensitivity, we employ t-SNE to visualize the latent representations produced during the encoding process. These representations are obtained from the output of ViT layers with and without CLP. For consistent comparison, we normalize the latent representation values based on the mean and standard deviation of both dimensions across voxels outside of BMs, thereby minimizing variability unrelated to tumor presence.

**Figure 7.**
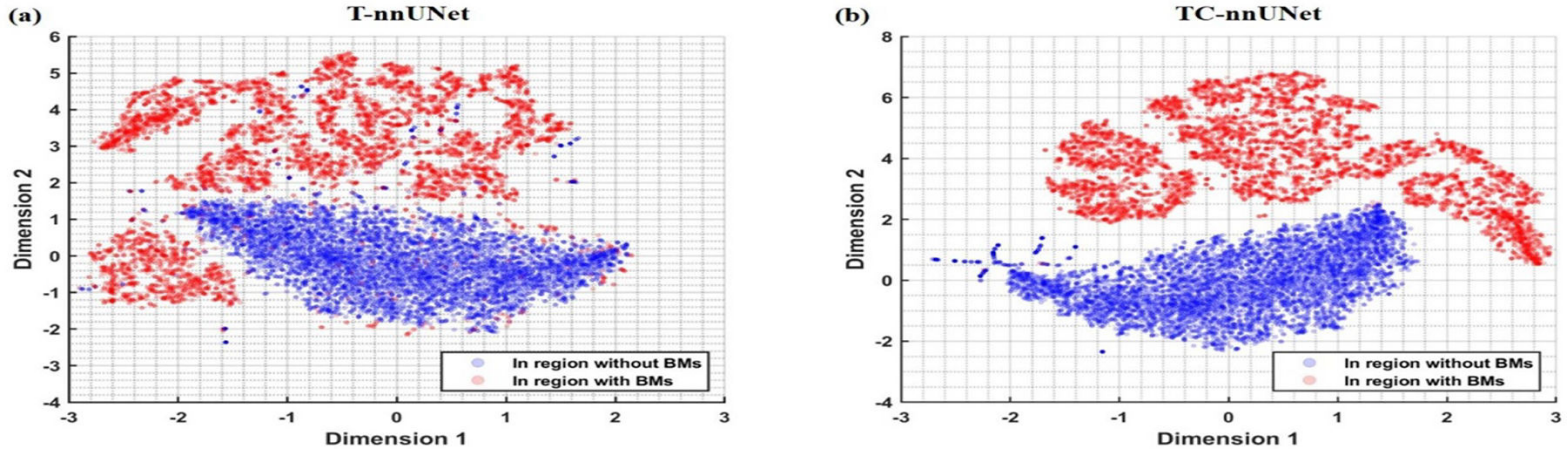
T-SNE visualization of voxel-wise latent representation after encoding process predicted by (a) T-nnUNet and (b) TC-nnUNet.

Figure 7 illustrates voxel-wise latent representation with and without CLP for T-nnUNet. In the CLP-enhanced TC-nnUNet model, the BMs latent representations (red markers) are consistently further from the non-BM representations (blue markers) compared to those in T-nnUNet model. This increased separation suggests that CLP encourages greater divergence between the latent features of BM and non-BM voxels, facilitating more distinct boundaries between classes. As a result, TC-nnUNet shows improved sensitivity and precision in BM detection (figure 3(a)), as the contrastive learning process enhances the model’s ability to distinguish BMs from surrounding tissue under a condition of longer distance in feature space.

## 4. Discussion

In this study, we developed and validated TLC-nnUNet for BMs detection and segmentation. This model achieved high precision and sensitivity for BMs detection and providing consistent, accurate contouring for BMs segmentation. By incorporating LRP and CLP, TLC-nnUNet yielded a sensitivity of 65.74% and a balanced precision of 81.61% for small BMs (<3 mm). At per patient level, the model achieves an overall a sensitivity of 89.70 ± 1.85%, a precision of 97.34 ± 0.77%, and a DC of 0.92 ± 0.06 representing a substantial improvement in accuracy compared to state-of-the-art methods. From a clinical standpoint, the improved sensitivity of TLC-nnUNet in detecting <3 mm BMs, while maintaining a low FP rate, is highly relevant to SRS, where treatment decisions depend heavily on precise lesion count and volume. In high-volume SRS practices, small or subtly enhancing lesions are frequently missed due to time constraints or their inconspicuous imaging features. By enhancing the detection of these lesions, TLC-nnUNet has the potential to reduce the risk of undertreatment and support more comprehensive, individualized SRS planning. That said, the clinical relevance of each detected lesion must still be assessed by the treating physician in the context of the patient’s overall condition, prognosis, and therapeutic goals. Further prospective clinical validation is needed to evaluate the model’s real-world impact on treatment planning, dosimetry, and patient outcomes.

One thing we would like to point out is that we employed a lesion-wise CV threshold of 0.5 as the primary matching criterion to distinguish between TP and FP detections. This choice was specifically made to prioritize segmentation fidelity alongside detection accuracy. By requiring at least 50% overlap, we ensure that a ‘detected’ lesion is represented by a robust segmentation rather than a trivial, single-voxel match, which would yield an unacceptably low DC and provide little clinical utility. We also clarify that because CV acts as a structural matching criterion rather than a network confidence score, its relationship with sensitivity and FP counts differs from traditional diagnostic thresholds. Specifically, lowering the CV threshold to broaden detection criteria would simultaneously increase the TP count and decrease the FP count (as more previously ‘unmatched’ predictions become ‘matches’). To maintain focus on clinically relevant detection and segmentations, we have reported our results at the established CV ≥ 0.5 operating point, which offers a balanced assessment of the TLC-nnUNet’s performance.

The ablation study across all BMs size at patient level and further analysis at BM level (detailed in sections 3.4 and 3.5, respectively) highlight the individual and combined impacts of LRP and CLP, underscoring the balanced trade-off between precision and sensitivity achieved by integrating both. Upon closer examination, adding T-nnUNet consistently outperforms the nnU-Net model (figure 3), particularly in identifying small or less conspicuous BMs (figure 5(a)), which are often challenging for traditional segmentation approaches. The LRP with a local-focused mechanism increases the model’s sensitivity to subtle BM features, which can be particularly beneficial for identifying small lesions. However, this heightened sensitivity occasionally leads to an increase in FP detections (figure 3(b)) with a misinterpretation of BMs (figure 5(b)), which is caused by the constraint of additional classification task on local region. In contrast, the CLP enhancement plays a complementary role by addressing the tendency for FP observed with LRP. CLP refines feature representations, diverging the representation of voxels with and without BMs, and improving detection sensing for elusive or low-contrast BMs compared to the original nnU-Net and baseline T-nnUNet configurations. Consequently, CLP contributes to the model’s ability to increase sensitivity while still enabling the model to maintain a high level of precision without sacrificing FP rates (figure 5).

In addition, we analyzed the weight of LRP (section 3.6), which provides deep interpretability into the decision-making process of T-nnUNet. We investigated how the weight of LRP in the loss function impacts detection precision and sensitivity. This analysis highlights a nuanced trade-off between sensitivity and precision as the LRP weight changes (figure 6). When the LRP weight is low, the model exhibits reduced sensitivity, often failing to detect small BMs. This limitation can be attributed to insufficient attention to fine details, resulting in missed detections and a high rate of FN, thereby lowering sensitivity. On the other hand, an excessively high LRP weight causes the model to become prone to FP. This shift occurs because stronger LRP weight biases the model toward predicting BM presence even in an ambiguous region, which will negatively influence the TP detection. Consequently, an overly high LRP weight negatively impacts precision by introducing more voxel-level FPs, which reduces model reliability. Selecting an optimal LRP weight is therefore essential for balancing the model’s ability to capture small BMs with high sensitivity while minimizing the risk of FPs. Based on our dataset, we determined that a moderate LRP weight of 0.4 provides the best compromise, enhancing sensitivity without incurring substantial losses in precision. This outcome underscores the importance of fine-tuning LRP weighting for a balanced detection model, especially when dealing with nuanced medical images where both FNs and FPs have clinical implications. Further studies are warranted to evaluate this balance across larger and more diverse datasets, which may provide insights into the stability and generalizability of the optimal LRP weight setting.

To better understand how the CLP enhances model performance in terms of precision and sensitivity, we conducted a detailed analysis using t-SNE to visualize the latent representations generated by the encoding layers (detailed in section 3.7). Figure 7 demonstrates that CLP enhances the model’s sensitivity and precision by limiting overlaps with non-BM features. By enlarging the contrastive distance between the two classes, CLP helps mitigate common classification challenges associated with BM detection, such as reducing FPs from ambiguous non-tumor areas and decreasing FNs where small or low-contrast BMs are overlooked. This increased separation in the latent space is crucial for voxel-wise classification during the decoding phase, as it enables the model to assign class labels more accurately based on the spatial and textural distinctions of BMs and non-BMs. The improvement directly translates to more reliable and accurate BMs detection outcomes, reducing FNs, which are critical for early and effective clinical intervention, and limiting FPs, which can otherwise lead to unnecessary SRS treatment. By structuring the latent space to more distinctly represent BM characteristics, CLP effectively optimizes model performance, balancing sensitivity and precision—a critical factor in applications requiring robust and interpretable results for accurate tumor detection and follow-up assessment as shown in figure 3(a). Beside BMs detection, inducing CLP into T-nnUNet or TL-nnUNet also highlights the value of contrastive learning in medical image segmentation proved by the increase of DC illustrated in figure 3(b). Therefore, voxel-wise CLP is able to benefit the object detection and segmentation related tasks in medical application.

Our future work will focus on applying this TLC-nnUNet to other medical applications involving the detection and segmentation of multiple small lesions, such as liver and lung metastases. In addition, to align with real-world SRS practice, the model ingests only a single T1c MRI image modality, which is generally adequate for brain metastasis detection and suits time-constrained workflows. The trade-off is reduced sensitivity to subtle contrast differences and a greater burden of FPs. Incorporating multi-modal inputs could improve detection of challenging cases. Finally, developing an adaptive LRP constraint that adjusts based on the shape, intensity contrast, and density of lesions can further improve sensitivity without compromising precision.

## Figures and Tables

**Table 1.** Performance comparison between TLC-nnUNet and current state-of-the-art methods for BMs detection and segmentation on T1c MRI, categorized by lesion size (longest axis length) at BM level.

Methods			Precision (%)			Sensitivity (%)			Mean DC	
CropNet(Dikici et al 2020)				—		—	—	90 (Mean 5 mm)	—	
SSD(Zhou et al 2020b)			100 (<3 mm)	35 ([3,6] mm)	36 (>6 mm)	15 (<3 mm)	70 ([3,6] mm)	98 (>6 mm)	—	
Fast R-CNN(Zhang et al 2020)			—			87 (size not reported)			—	
En-DeepMedic(Liu et al 2017)			—			—			0.67 (size not reported)	
Fast R-CNN(Xue et al 2020)			—			—			0.89 (size not reported)	
MetNet(Zhou et al 2020a)			58 (1–52 mm)		67 (>6 mm)	88 (1–52 mm)		99 (>6 mm)	0.85 (1–52 mm)	0.87 (>6 mm)
Asymmetric UNet(Cao et al 2021)			72 (1–10 mm)		82 (11–26 mm)	76 (1–10 mm)		94 (11–26 mm)	0.65 (1–10 mm)	0.84 (11–26 mm)
Self-adaptive nnU-Net(Ziyaee et al 2023)			90 (1–63 mm)		96 (>6 mm)	88 (1–63 mm)		95 (>6 mm)	0.82 (1–63 mm)	0.87 (>6 mm)
CAD(Fairchild et al 2023)			59 (<3 mm)			94 (<3 mm)			0.79 (<3 mm)	
DeSeg(Yu et al 2023)			77 (<1.5cc)			91 (<1.5cc)			0.86 (>1.5cc)	
TLC-nnUNet (Ours)	Training	Testing	<3 mm	[3,6] mm	>6 mm	<3 mm	[3,6] mm	>6 mm	3–48 mm	
	Pooled data BraTS(199) + UTSW (203)	BraTS + UTSW(79)	82	92	99	66	88	94	0.90	
		UTSW (40)	85	92	99	64	91	95	0.91	
		BraTs (39)	81	92	99	67	86	93	0.88	
	BraTS (238)	UTSW (243)	87	92	99	63	93	96	0.91	
	UTSW (243)	BraTS (238)	81	92	99	68	84	92	0.87	

*Note:* Results for baseline models are cited directly from their original publications; note that datasets and evaluation protocols may vary across these sources.

## Data Availability

The data cannot be made publicly available upon publication due to legal restrictions preventing unrestricted public distribution. The data that support the findings of this study are available upon reasonable request from the authors.
